# In Vivo Determination of the Human Corneal Elastic Modulus Using Vibrational Optical Coherence Tomography

**DOI:** 10.1167/tvst.11.7.11

**Published:** 2022-07-13

**Authors:** Marcos A. Crespo, Hiram J. Jimenez, Tanmay Deshmukh, Jose S. Pulido, Ahmed Saeed Saad, Frederick H. Silver, Dominick A. Benedetto, Christopher J. Rapuano, Zeba A. Syed

**Affiliations:** 1 Cornea Service, Wills Eye Hospital, Sidney Kimmel Medical College at Thomas Jefferson University, Philadelphia, PA, USA; 2 Vickie and Jack Farber Vision Research Center, Wills Eye Hospital, Philadelphia, PA, USA; 3 OptoVibronex, LLC, Bethlehem, PA, USA; 4 Department of Pathology and Laboratory Medicine, Robert Wood Johnson Medical School, Piscataway, NJ, USA; 5 Center for Advanced Eye Care, Vero Beach, FL, USA

**Keywords:** cornea, biomechanics, elastic modulus, vibrational OCT, mechanical properties

## Abstract

**Purpose:**

To determine the in vivo elastic modulus of the human cornea using vibrational optical coherence tomography (VOCT).

**Methods:**

Vibrational analysis coupled with optical coherence tomography (OCT) was used to obtain the resonant frequency (RF) and elastic modulus of corneal structural components. VOCT corneal thickness values were measured using OCT images and correlated with corneal thickness determined with Pentacam (Oculus, Wetzlar, Germany). Moduli were obtained at two locations: central cornea (CC) and inferior cornea (IC). Measurements were obtained with and without anesthetic eye drops to assess their effect on the modulus measurements.

**Results:**

VOCT thickness values correlated positively (*R*^2^ = 0.97) and linearly (y = 1.039x–16.89) with those of Pentacam. Five RF peaks (1–5) were present, although their presence was variable across eyes. The RF for peaks 1 to 5 in the CC and IC ranged from 73.5 ± 4.9 to 239 ± 3 Hz and 72.1 ± 6.3 to 238 ± 4 Hz, respectively. CC and IC moduli for peaks 1 to 5 ranged from 1.023 ± 0.104 to 6.87 ± 0.33 MPa and 0.98 ± 0.15 to 6.52 ± 0.79 MPa, respectively. Topical anesthesia did not significantly alter the modulus (*P* > 0.05 for all), except for peak 2 in the CC (*P* < 0.05).

**Conclusions:**

This pilot study demonstrates the utility of VOCT as an in vivo, noninvasive technology to measure the elastic modulus in human corneas. The structural origin of these moduli is hypothesized based on previous reports, and further analyses are necessary for confirmation.

**Translational Relevance:**

This work presents VOCT as a novel approach to assess the in vivo elastic modulus of the cornea, an indicator of corneal structural integrity and health.

## Introduction

The unique physical properties of the human cornea, including clarity, distinctive hydration, and mechanical strength, are the result of its highly organized structure.^1^^–^^4^ Alterations in biomechanical properties of the cornea occur in degenerative diseases such as keratoconus, connective tissue diseases like Marfan disease, and iatrogenic corneal ectasia.^1^^,^^5^^,^^6^ Widespread interest in the measurement of corneal biomechanical properties in vivo has promoted the development of instrumentation for clinical application such as the Ocular Response Analyzer (ORA; Reichart Ophthalmic Instruments, Buffalo, NY, USA),^7^^,^^8^ Corvis ST (Oculus, Wetzlar, Germany),^9^^,^^10^ Brillouin optical microscopy,^11^^,^^12^ magnetic resonance elastography,^13^^,^^14^ and optical coherence elastography.^15^^–^^18^

Vibrational optical coherence tomography (VOCT) is a novel approach that utilizes optical coherence tomography (OCT) to measure the resonant frequency (RF) of corneal components excited by a spectrum of audible sound frequencies. Through a calibration graph, RFs are converted to elastic moduli.^19^^,^^20^ While the RF depends on corneal thickness, the elastic modulus is independent of thickness.^19^^,^^20^

To date, VOCT has been used in vitro to measure the modulus of decellularized dermis, essentially collagen, with results similar to those measured by standard tensile stress–strain testing, suggesting that VOCT is comparable to widely accepted testing modalities.^19^ In addition to skin, VOCT has been used to measure the biomechanical properties of arteries, tendons, nerves, muscle, and the macromolecular components of extracellular matrices (ECMs).^20^^,^^21^ In vitro measurements of the tissue modulus of human and porcine corneas by VOCT are reported to be about 2 MPa.^22^ In this pilot study, we report the in vivo use of VOCT to determine the elastic moduli of the corneas of healthy human volunteers.

## Methods

### Patient Selection

This prospective study complied with the Health Insurance Portability and Accountability Act, adhered to the tenets of the Declaration of Helsinki, and was approved by the Wills Eye Hospital Institutional Review Board (IRB#2021-36). All participants provided informed consent prior to inclusion in the study.

A total of 21 adult participants at Wills Eye Hospital were recruited, of whom 16 (32 eyes) met the inclusion criteria to participate in this study. Inclusion criteria included 21 years of age or older, no history of refractive or other corneal surgery, no evidence of ectasia as determined by Pentacam (Oculus), a normal slit-lamp examination, and a best-corrected Snellen visual acuity of 20/30 or better in both eyes. Patients were excluded if they had a final Pentacam D-value greater than or equal to 1.6 in either eye, had diabetes, or had any connective tissue disorder such as Marfan syndrome, Ehlers-Danlos syndrome, or osteogenesis imperfecta.

### Demographic and Clinical Data Collection

Demographic data (age and sex) were collected for each participant. Pentacam scans (4-Map Refractive and Belin/Ambrosio Enhanced Ectasia Display) of each eye were reviewed to rule out any evidence of corneal ectasia. Central corneal thickness (CCT) for each eye was acquired using the Pentacam.

### Data Allocation for the Correlation Process and Experimental Modulus Assessment

Demographic and clinical data were collected by three authors (MAC, HJJ, ZAS). VOCT acquisition and analysis were performed by separate authors masked to the aforementioned data (TD, FHS) to reduce bias and preserve the objectivity of postimaging processing. Pentacam thickness values from four eyes of two participants were disclosed to the VOCT acquisition team to be used for system calibration. Postcalibration thickness values from the remaining 28 eyes were compared to Pentacam values to assess the reliability of VOCT thickness values.

### VOCT In Vivo Elastic Modulus Measurements

**Figure 1.**
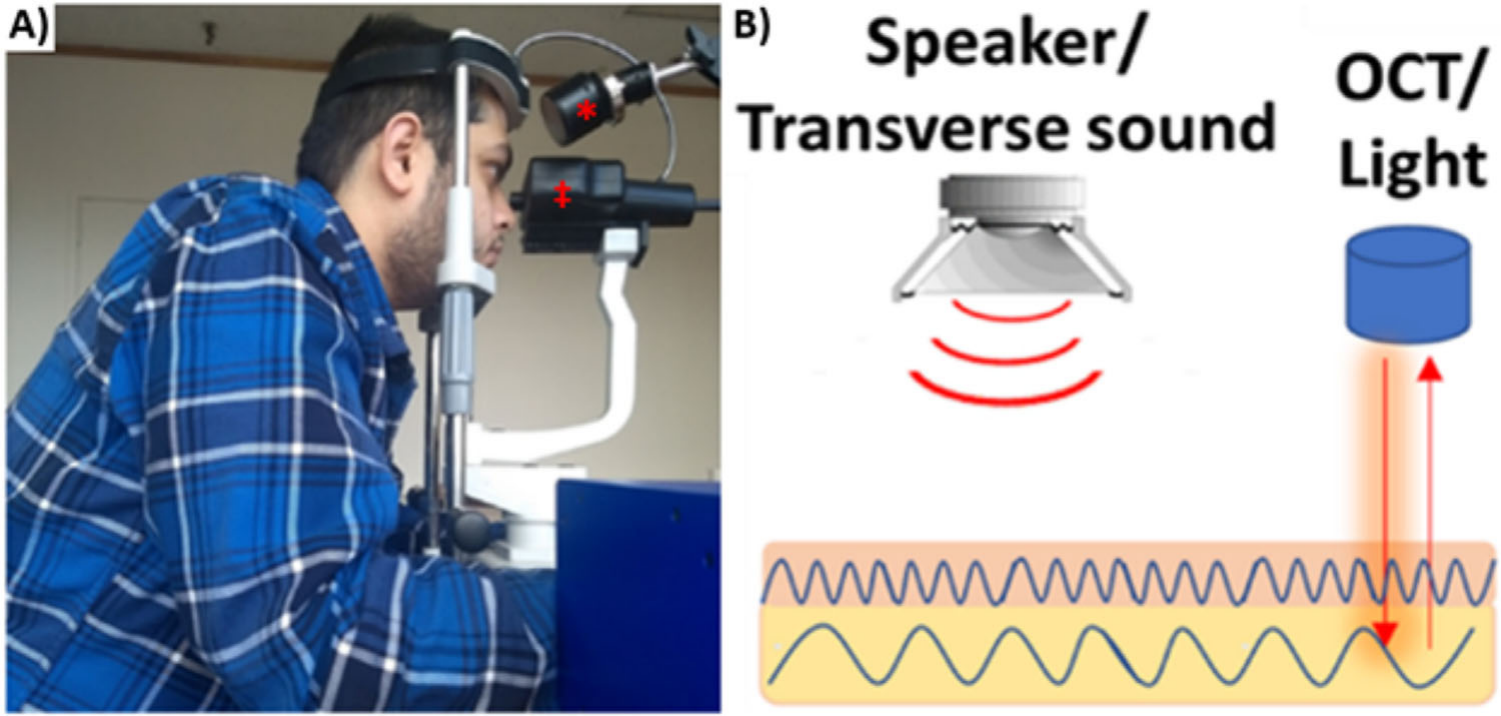
(A) In vivo VOCT setup showing both the OCT device (‡) and speaker (*). (B) Schematic representation of the VOCT mechanism: transverse sound waves are emitted by a speaker, producing tissue vibrations detected as displacement by the OCT device.

Patients were positioned in front of the OCT (Lumedica, Durham, NC, USA) with the acoustic Bluetooth speaker (EWA A106 Pro; J.Y.M. Digital Technology Co., Ltd, Shenzhen, Guangdong, China) placed near the eye undergoing testing, although with no direct ocular contact (Fig. 1A). A frequency-generating application capable of driving the speaker between 30 and 20,000 Hz was downloaded onto the i5 processor within the OCT device to produce the sound waves. A spectral-domain OCT system that uses a fiber-coupled superluminescent diode light source with an 840-nm center wavelength and a 100-nm bandwidth (full width at half maximum) operating on A-mode was used to detect the transverse displacement in an area of approximately 0.25 mm, as previously described (Fig. 1B).^21^

The RF at two locations, the central cornea (CC) and inferior cornea (IC), were determined by measuring the displacement of the cornea resulting from driving sinusoidal frequencies ranging from 50 to 250 Hz, in 10-Hz steps. Central measurements were taken from the visual axis, and participants would focus directly on a centrally located small red dot generated by the OCT device. For inferior measurements, the patient would focus on a fixed point superiorly on the ceiling of the testing room. Using the camera coupled to the VOCT device, we would locate a point inferiorly at the 6-o'clock position, roughly 2 to 3 mm from the inferior limbus. These points were selected because they could be easily duplicated in different patients and were far enough away from the eyelid and eyelashes to avoid interference from these structures. A period of 2 seconds was allowed between each measurement to permit total corneal relaxation before subsequent measurements. The weighted displacement of the sample was obtained by dividing the displacement observed at a frequency by the displacement measured in the absence of the sample. Weighted displacement versus frequency data provided a vibrational spectrum generated by various components of the corneal tissue. The RF was defined as the frequency in which the raw OCT image demonstrated the greatest transverse displacement in phase with the inputted sinusoidal sound wave, which would correspond to an RF peak on the vibrational spectrum. RF peaks were considered significant if they were equal to or greater than 30% of the highest RF observed within the vibrational spectrum, excluding marginal frequencies (50 and 250 Hz).

The resulting RFs of the CC and IC were then converted into values of elastic modulus (MPa) using a calibration equation described in previous studies^23^:

$$\begin{array}{c} E*d=0.0651*\left(fn^{2}\right)+233.16, \end{array}$$

where E represents the elastic modulus, d is the corneal thickness obtained from OCT imaging, and fn^2^ is the square of the RF. This equation was developed from previously published results of in vitro analyses of different human and porcine soft tissues using uniaxial tensile testing and VOCT analysis, as well as in vivo measurements on human skin.^19^^,^^22^^,^^24^^,^^25^ This equation is employed under the assumption that most body soft tissues have a density close to 1.0.^26^

### VOCT Measurements With Topical Anesthesia Drops

Modulus values before and after the use of anesthesia were compared to assess the agreement of the VOCT measurements with the use of anesthesia. To assess the effect of topical anesthesia drops on VOCT measurements, testing was repeated on 10 eyes of 10 patients at both locations (CC, IC) 1 to 2 minutes after administering one drop of topical anesthesia (proparacaine hydrochloride ophthalmic solution USP, 0.5%) bilaterally. The patients were instructed to blink three times between each sound frequency measurement to help maintain corneal hydration.

### Statistical Analysis

Descriptive statistics were used for baseline demographic characteristics and VOCT results. For continuous numerical values, we used Student's *t*-test to determine significant differences between the groups. Univariate linear regression analysis was employed to explore the relationship between corneal thickness measurements taken from VOCT and those from Pentacam. *P* values less than 0.05 were considered significant. Box-and-whisker plots were used to illustrate the means, quartiles, and upper and lower limits of elastic modulus values for each RF peak as well as to compare values before and after the use of topical anesthesia. Bland–Altman plots were used to display the agreement between modulus values before and after topical anesthesia drops. Statistical analyses were performed with SPSS 28.0 (IBM Corp., Armonk, NY, USA).

## Results

### Demographic Data

Sixteen participants underwent VOCT testing. The mean age for the study group was 34.0 ± 9.4 years (range: 23–54 years). Ten of the participants were male (62.5%) and six were female (37.5%).

### Linear Regression Analysis of VOCT to Pentacam Thickness Values

**Figure 2.**
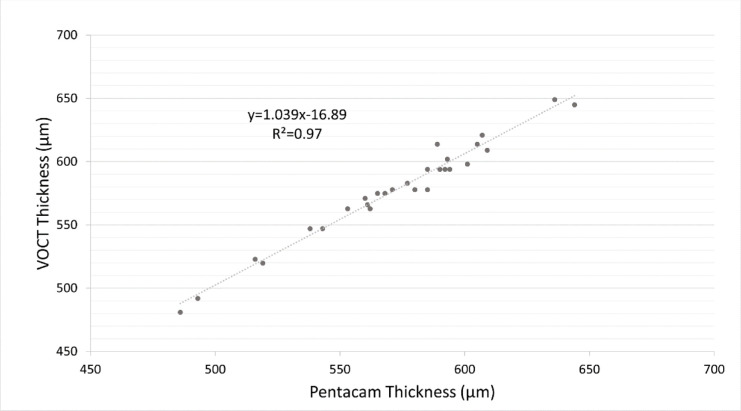
Linear regression comparing VOCT and Pentacam central corneal thickness measurements showed a strong correlation between the two thickness measurements.

Pentacam CCT values from four eyes were used to calibrate the VOCT machine. Postcalibration VOCT CCT measurements from the remaining 28 eyes were compared to their respective Pentacam values and showed a positive (*R*^2^ = 0.97) and linear correlation (y = 1.039x–16.89) (Fig. 2).

### Resonant Frequency and Elastic Modulus Values

**Figure 3.**
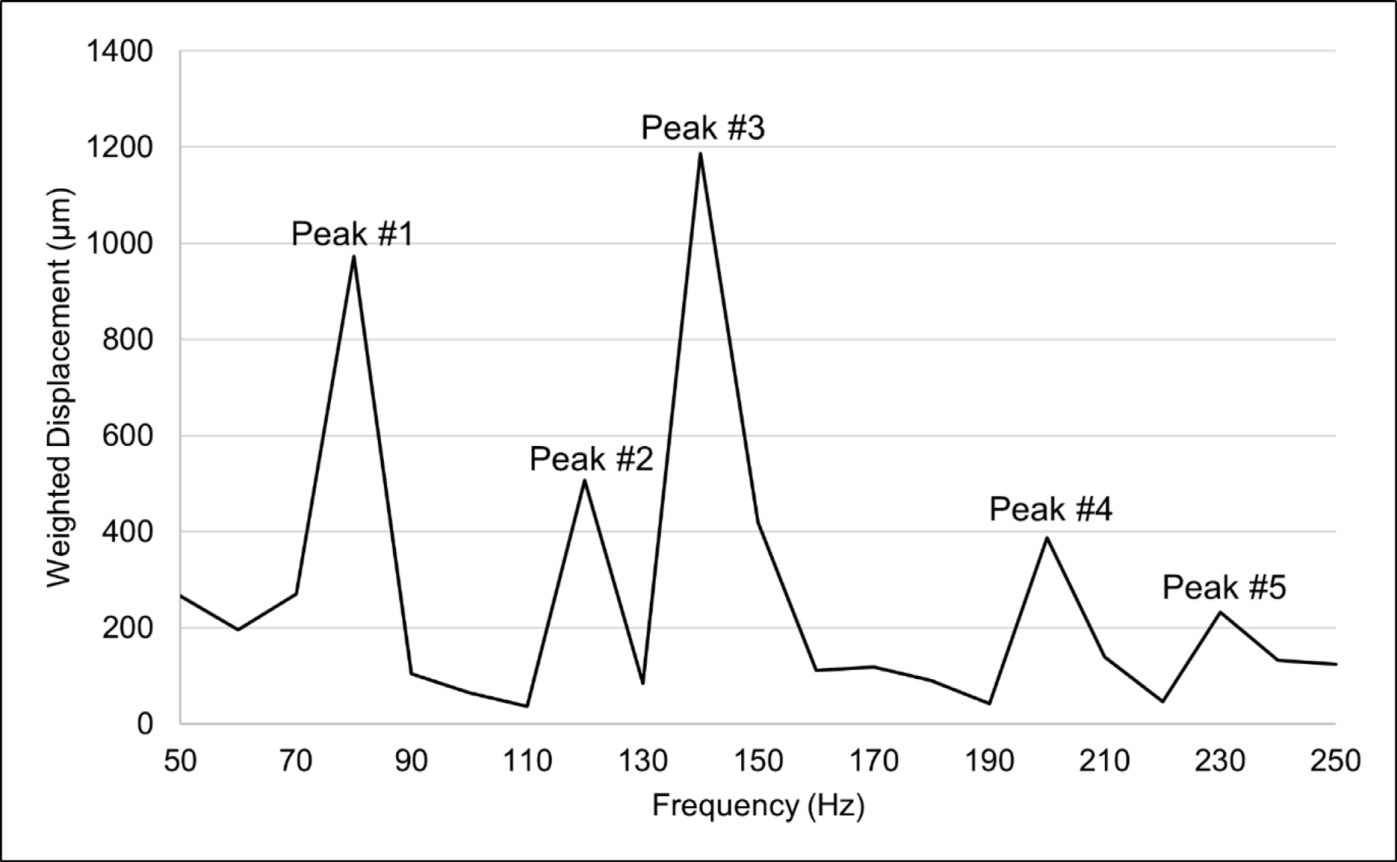
Representative output of frequency spectrum versus weighted displacement displaying resonant frequency peaks 1 to 5.

Five discrete RF peaks (1–5) were identified in our analysis (Fig. 3). The means and standard deviations of the RFs for the CC and IC are shown in Table 1. In addition to peaks 1 to 5, a small percentage of patients presented other variable peaks (Supplementary Fig. S1).

**Table 1.** Resonant Frequency and Moduli Values for Peaks 1 to 5 for Central and Inferior Cornea

	Central Cornea		Inferior Cornea	
Peak	Resonant Frequency (Hz), Mean ± SD	Modulus (MPa), Mean ± SD (95% CI)	Resonant Frequency (Hz), Mean ± SD	Modulus (MPa) Mean ± SD, (95% CI)
1	73.5 ± 4.9	1.023 ± 0.104	72.1 ± 6.3	0.98 ± 0.15
		(1.009–1.037)		(0.96–0.99)
2	120.4 ± 2.0	2.05 ± 0.16	120.3 ± 1.8	1.991 ± 0.236
		(2.03–2.07)		(1.961–2.020)
3	148.7 ± 8.0	2.94 ± 0.40	147.2 ± 6.7	2.76 ± 0.28
		(2.89–3.00)		(2.71–2.81)
4	207 ± 7	5.31 ± 0.37	205 ± 6	5.08 ± 0.73
		(5.23–5.40)		(4.95–5.22)
5	239 ± 3	6.87 ± 0.33	238 ± 4	6.52 ± 0.79
		(6.81–6.93)		(6.40–6.65)

CI, confidence interval.

**Figure 4.**
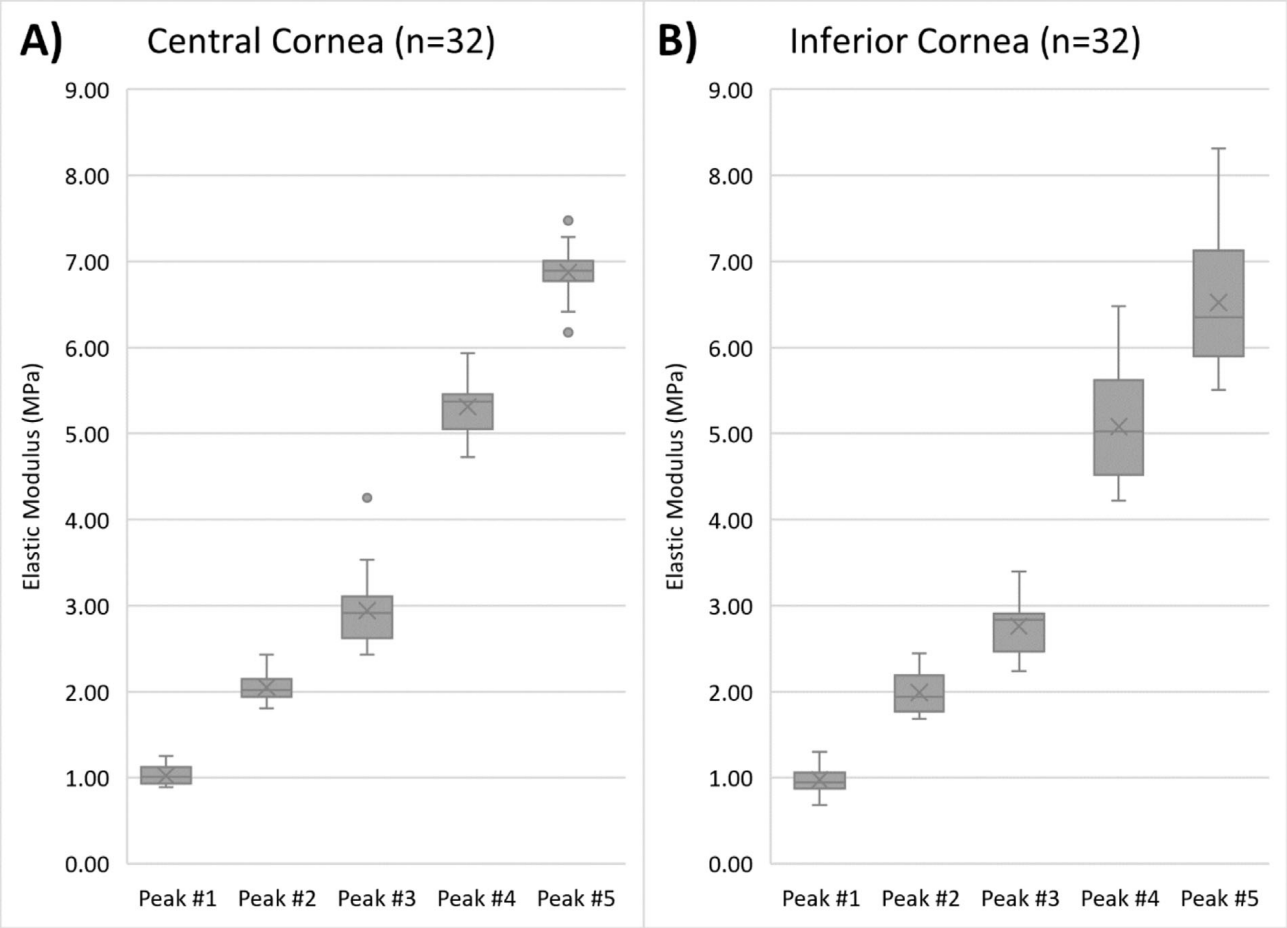
Elastic modulus values for resonant frequency peaks 1 to 5 for (A) central and (B) inferior cornea in 32 eyes. The *horizontal lines* in the box-and-whisker plots represent the median values, and the *boxes* represent the lower and upper quartiles. The *x* represents the mean, and the *bars* represent minimum and maximum values within 1.5 times the lower and upper quartiles. The *dots* represent the outlier values.

The elastic modulus was determined using the calibration equation, the thickness, and RF values obtained from VOCT (Fig. 4). Elastic modulus values for peaks 1 to 5 in CC and IC are presented in Table 1. Mean modulus values for the peaks ranged from 1.023 to 6.87 MPa in the CC and 0.98 to 6.52 MPa in the IC.

### Effect of Topical Anesthesia on Corneal Thickness and Elastic Modulus Values

Ten patients underwent repeat VOCT testing on one eye at both locations after placement of topical anesthesia. Mean thickness measurements for the CC showed a significant decrease from 591 to 557 µm (*P* < 0.05), although the percentage difference was small (5.8%). For IC, mean thickness values decreased from 628 to 614 µm, although the change was not significant (*P* = 0.47). CC elastic modulus values for peaks 1 and 3 to 5 had no significant difference (*P* > 0.05 for all), while peak 2 had a significant difference (*P* < 0.05). The percentage difference was small (7.3%), changing from 1.985 MPa without anesthesia to 2.13 MPa with anesthesia in a subset of 10 eyes. The IC modulus calculated after the use of anesthesia did not differ significantly for all peaks (*P* > 0.05 for all). A comparison of modulus values before and after the use of anesthesia for CC and IC is shown in Supplementary Figure S2. Furthermore, measurements for modulus values showed acceptable agreement as seen in the Bland–Altman plots, where most values fell within the 95% confidence interval (Supplementary Fig. S3). A sample effect size analysis between the preanesthesia and postanesthesia measurements for peaks 1 to 5 was performed. The sample effect size for the central cornea was small for peaks 1, 3, and 5 and large for peaks 2 and 4. In the inferior cornea, the sample effect size was trivial for peak 1, small for peak 2, medium for peak 3, and large for peaks 4 and 5.

## Discussion

In this pilot study, VOCT was employed to measure the RFs and elastic moduli values of normal human corneas in vivo. For the first time, five corneal RF peaks (peaks 1–5) were identified, although their presence was variable across eyes (Supplementary Fig. S1). Elastic moduli of the CC ranged from 1.023 to 6.87 MPa, while the IC elastic moduli ranged from 0.98 to 6.52 MPa. Each peak appears to correspond to different structural components within the cornea. Although further analyses are needed to identify the specific sources of each peak, we can hypothesize the origins. For example, peak 1 may correspond to the cellular component of the cornea since a similar RF of around 50 to 70 Hz has been previously correlated to the cellular section of the epidermis.^26^ The remaining peaks 2 to 5 likely correspond to different collagen networks within the stroma, the main contributor to the cornea's structural integrity and mechanical strength.^6^^,^^27^ These networks may vary in stiffness and orientation depending on the location in the cornea, which may explain the presence of several unique peaks.^22^^,^^27^^,^^28^ Results from previous VOCT studies also suggest that the modulus of peak 1 (approximately 1 MPa) may correspond to the cellular components in the cornea and the modulus of peaks 2 to 5 (approximately 2 to 7 MPa) to the fibrillar collagen in the lamellae and larger collagen fibers.^22^^,^^26^

Age-dependent variability in moduli values was expected in the process of corneal biomechanical analysis, as reported by other authors.^29^ However, due to the limited age range of participants in our study, we could not confirm this observation using VOCT. Future studies designed toward this end could be performed to analyze this phenomenon further.

Calculating the elastic modulus requires a value for corneal thickness. The reliability of thickness measurements obtained by the VOCT device was confirmed by the strong correlation between measurements taken with the Pentacam and VOCT.

We also explored the effect of anesthetic drops on VOCT measurements, as topical anesthesia improved patient comfort and fixation during the measurement process. Some authors have found that corneal hydration plays a role in measured modulus values,^30^^–^^32^ and others have concluded that anesthesia may lead to changes in corneal hydration levels.^33^^,^^34^ Thus, we considered it necessary to explore the possibility of a change in modulus resulting from alterations in hydration related to anesthetics. VOCT thickness before and after anesthesia was significantly different for the CC (*P* < 0.05), although the percentage difference was small (5.8%). Thickness in the IC did not differ significantly. This difference may be attributed to changes in corneal hydration caused by the use of anesthesia. No statistical significance was found for the modulus values before and after employing anesthesia, except for peak 2 in the CC, in which the difference was significant (*P* < 0.05). Nevertheless, the percentage difference was minor (7.3%). Since the small sample size limits the statistical power of our findings, a larger cohort of patients could be studied in the future to understand the changes that occur after the administration of anesthesia. Sample effect size analysis showed variable impact depending on the peak and location measured. We expect that future studies with larger cohorts may strengthen these results.

The biomechanical properties of the cornea are challenging to assess due to tissue heterogeneity, anisotropy, nonlinearity, and viscoelasticity.^35^^–^^40^ Traditionally, corneal biomechanical measurements have been performed via uniaxial extension tests, where a strip of corneal tissue is analyzed in vitro to assess its stress–strain relationship and provide an elastic modulus value.^41^ However, this method cannot be employed clinically since it is destructive and poorly mimics in vivo properties such as humidity, geometry, and anatomical relationships. There is current interest in identifying nondestructive in vivo approaches to measure elastic modulus. In addition to VOCT, methods being utilized to determine corneal biomechanics include ORA,^7^^,^^8^ Corvis ST,^9^^,^^10^ Brillouin optical microscopy,^11^^,^^12^ magnetic resonance elastography,^13^^,^^14^ and optical coherence elastography^15^^–^^18^ (Table 2).

**Table 2.** Selected Methods for Corneal Biomechanical Analysis

Methods	Corneal Biomechanical Property	Values	Measurement Approach	Limitations and Assumptions
Uniaxial extension tests (extensiometry)	Elastic modulus	0.8–2.2 MPa^41^	A strip of corneal tissue is stretched while analyzing stress–strain relationship.^41^	Destructive.^41^
Ocular response analyzer	Hysteresis	9.6 mm Hg^7^	A high-speed air puff is delivered to the cornea, provoking a transient inward deformation of the cornea. An infrared beam that reflects from the cornea to a detector can detect the inward and outward deformation of the cornea; coupled with the pressure measurement at both deflections, a hysteresis value is determined.^49^	Measurements are dependent on geometry and IOP.^50^ Different combinations of viscous damping and elastic modulus can produce the same hysteresis values.^51^
Corvis ST	Elastic modulus	0.71 MPa^52^ 0.207 MPa^53^	A puff of air is applied to deform the cornea. The resulting corneal oscillations are observed using an ultra-high-speed camera. Computational algorithms are then able to process the images and determine different parameters.^9^	Measurements are dependent on geometry and intraocular pressure (IOP).^50^ Assumes isotropy and linearity in viscoelasticity.^52^
Brillouin optical microscopy	Elastic modulus	2.7 GPa^11^	The spectral shift in Brillouin light scattering is measured, which is proportional to the longitudinal elastic modulus of the tissue. Brillouin optical microscopy allows for the measurement of corneal elastic modulus at different depths.^11^	Measurements are susceptible to mass density, refractive index, and hydration levels.^54^
Magnetic resonance elastography	Elastic modulus	40–185 kPa^13^	Magnetic resonance imaging is used to analyze tissue motion upon the application of mechanical vibrations. The phase shift observed is employed to calculate the elastic modulus.^14^	Assumes isotropy, homogeneity, and almost complete incompressibility.^13^
Optical coherence elastography	Elastic modulus	11.52 kPa^55^	Light is used to examine tissues based on the amount of reflected and nonreflected light an image produces.^17^ Corneal displacement measurements are obtained while producing small displacements utilizing mainly a pushing element^17^^,^^55^ or an air puff.^18^ Mathematical calculations can then be performed to determine the elastic modulus of tissues.^55^	Assumes linear elasticity,^56^ incompressibility,^56^ isotropy,^57^ and homogeneity.^57^ In the applications that employ an air puff, the measurements are dependent on geometry and IOP.^50^
Vibration optical coherence tomography (VOCT)	Elastic modulus	0.98–6.87 MPa (This article)	Noninvasive and nondestructive approach that employs infrared light and audible sound to create transverse tissue displacement, which results in a spectrum of resonant frequencies that are recorded by measuring the displacement of the tissue as a function of frequency. The measured resonant frequencies are converted into elastic modulus values using a calibration equation.^26^	Modulus calculation is dependent on the reliability of the calibration equation. The RF value obtained is precise within a ± 10-Hz range. Assumes a density near 1.0.^26^

Currently, the ORA is the most commonly used clinical device that has been inferred to measure corneal biomechanical properties.^42^ The ORA measures hysteresis, which weakly correlates with corneal thickness and is a measure of the difference in the deformation that occurs during loading and unloading of the cornea.^7^ Alterations in hysteresis can be observed in some conditions that affect the cornea.^7^ For example, corneal ectasia is associated with reduced hysteresis, but currently, these measurements cannot differentiate normal corneas from those with mild keratoconus.^8^ Hysteresis values could be considered a surrogate measurement for changes in corneal deformation under a fixed load, although it is not a direct measurement of corneal stiffness since stiffness is related to the tangent to the force-deformation curve. Thus, the clinical use of hysteresis is limited, and comparison can only be drawn to standard mechanical tests that measure changes in recovery of the corneal thickness after a load is removed.

Unlike hysteresis, the other methods mentioned above can estimate elastic modulus, although they differ greatly in magnitude (kPa-GPa) and numerical value. This broad range can be attributed to the assumptions and limitations made in their respective modulus calculations (Table 2). The assumptions of incompressibility (Poisson's ratio of 0.5) and time-independence stand out as a difference from our method. Poisson's ratio for soft tissues (dermis) has been documented to range from 0.38 to 0.63, which suggests that ECMs do not deform at constant volume, thus implying that methods that assume incompressibility are prone to errors in the calculation process.^21^ Among the techniques discussed, Brillouin optical microscopy determines the modulus value with the highest order of magnitude. This is mainly due to the time scale of pressure modulation, which is measured in GHz for acoustic phonons as opposed to Hz that are characteristic of mechanical stress. Since higher frequencies lead to a decrease in relaxation time, a higher modulus is observed.^11^^,^^43^ These differences also occur since the methods are not calibrated against any standard mechanical testing procedure, a unique characteristic of VOCT.

VOCT features several qualities that make it particularly advantageous for the clinical assessment of corneal modulus. VOCT is nondestructive, as opposed to classical uniaxial stress–strain measurements.^41^ VOCT can provide information on regional variability of corneal biomechanics that may be required for clinical applications such as early detection of keratoconus while current commercially available methods (ORA^7^ and Corvis ST^17^) lack spatial resolution. VOCT does not require assuming a Poisson's ratio to calculate the elastic modulus.^22^ VOCT has a very high resolution and is noncontact, as opposed to magnetic resonance elastography (MRE) and optical coherence elastography.^17^ Unlike other methods, VOCT provides elastic modulus values in the order of magnitude (MPa) that are commonly reported on standard stress–strain tests. Other authors have explored a similar method employing vibrational analysis coupled with OCT measurements on bovine and porcine corneas but do not consider the thickness measurements required for the appropriate determination of RF and, in turn, corneal elastic modulus.^44^^,^^45^ Additionally, these studies used sound volumes around 100 dB, which are not safe for human use. In contrast, the VOCT technology described in this report employs sounds of around 50 dB.

The clinical utility of VOCT may be augmented with additional innovations. Eye-tracking technology can be implemented to reduce the chances of fixation loss during measurements. The process of obtaining four VOCT measurements (i.e., two in each eye) takes approximately 20 to 30 minutes total. Decreasing testing time would permit better in-clinic time management. The VOCT machine acquires elastic modulus values for the entire depth of a single location on the cornea, and the analysis is made in a small area of the cornea of around 0.25 mm in diameter. Acquiring a depth-dependent analysis of the modulus in multiple locations would further benefit the clinician as it would offer a better picture of the overall corneal biomechanical characteristics.

Our study has several limitations, such as the small sample size. Future studies with a larger number of patients will help substantiate our current findings. Regarding the precision of the measurements, RF determination has a margin of error of ±10 Hz, and the reliability of the RF and elastic modulus measurements performed by location could be impacted if any change in fixation occurred during the measurement process. The main limitation of the study is that currently, there is no standard technique to assess in vivo corneal modulus. Thus, the elastic modulus values obtained via VOCT in this study cannot be validated against other in vivo methods, but our measurements were well in line with mechanical measurements of similar viscoelastic substances and ex vivo corneal measurements that are in the range of 0.8 to 57 MPa.^41^^,^^46^^–^^48^ Finally, we currently cannot accurately determine which layers of the cornea produce each RF peak. Future histopathologic and ex vivo keratectomy studies using VOCT will help us identify the corresponding layers.

In conclusion, this pilot study demonstrates the feasibility of utilizing VOCT to assess corneal RFs and elastic moduli. The noninvasive characteristic of the VOCT measurements makes it ideal for clinical applications. Future studies could employ VOCT to characterize corneal pathologies such as keratoconus and postrefractive surgery ectasia to further assess the method's utility in the clinical setting.

## Supplementary Material

Supplement 1

Supplement 2

Supplement 3
