# Supplementary material for: In Vivo Determination of the Human Corneal Elastic Modulus Using Vibrational Optical Coherence Tomography
Source: Transl Vis Sci Technol. 2022 Jul 13;11(7):11. doi: 10.1167/tvst.11.7.11 (PMC9288150; doi:10.1167/tvst.11.7.11)
Supplement: Supplement 1 [file tvst-11-7-11_s001.pdf]

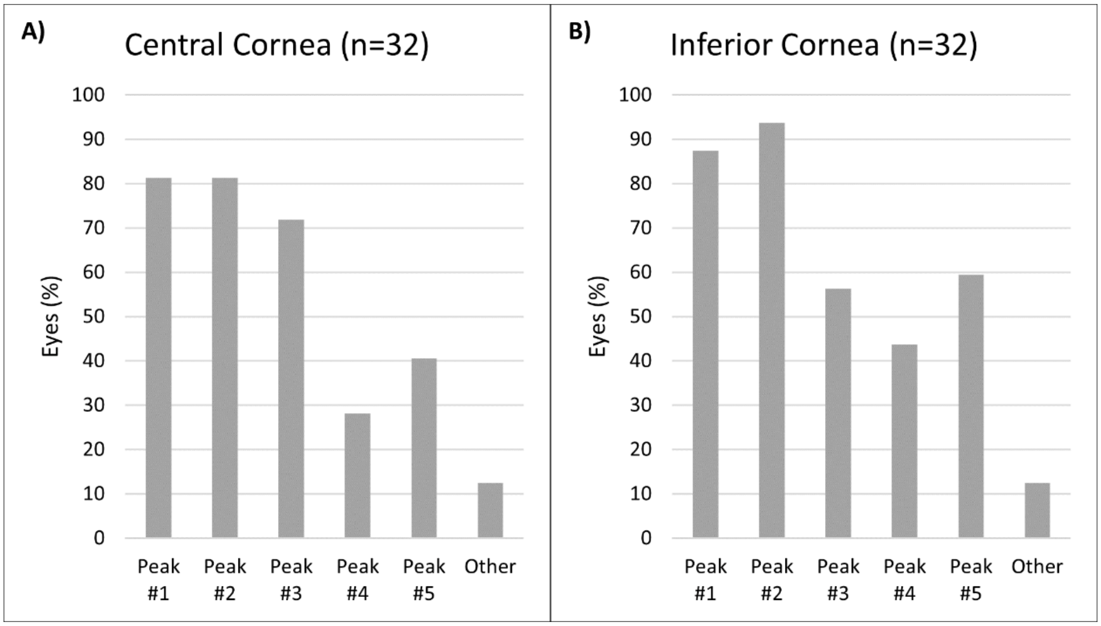

Figure S1. Distribution of resonant frequency peaks for (A) central and (B) inferior cornea. Peak #1 was present in 81.3% (n=26) of CC and 87.5% (n=28) of IC measurements. Peak #2 was present in 81.3% (n=26) of CC and 93.8% (n=30) of IC measurements. Peak #3 was present in 71.9% (n=23) of CC and 56.3% (n=18) of IC measurements. Peak #4 was present in only 28.1% (n=9) and 43.8% (n=14) of CC and IC measurements, respectively. Peak #5 was present in 40.6% (n=13) and 59.4% (n=19) of CC and IC measurements, respectively. Peaks #1-3 were present concomitantly in 53.1% (n=17) of CC and 53.1% (n=17) of IC measurements.
