# Supplementary material for: In Vivo Determination of the Human Corneal Elastic Modulus Using Vibrational Optical Coherence Tomography
Source: Transl Vis Sci Technol. 2022 Jul 13;11(7):11. doi: 10.1167/tvst.11.7.11 (PMC9288150; doi:10.1167/tvst.11.7.11)
Supplement: Supplement 2 [file tvst-11-7-11_s002.pdf]

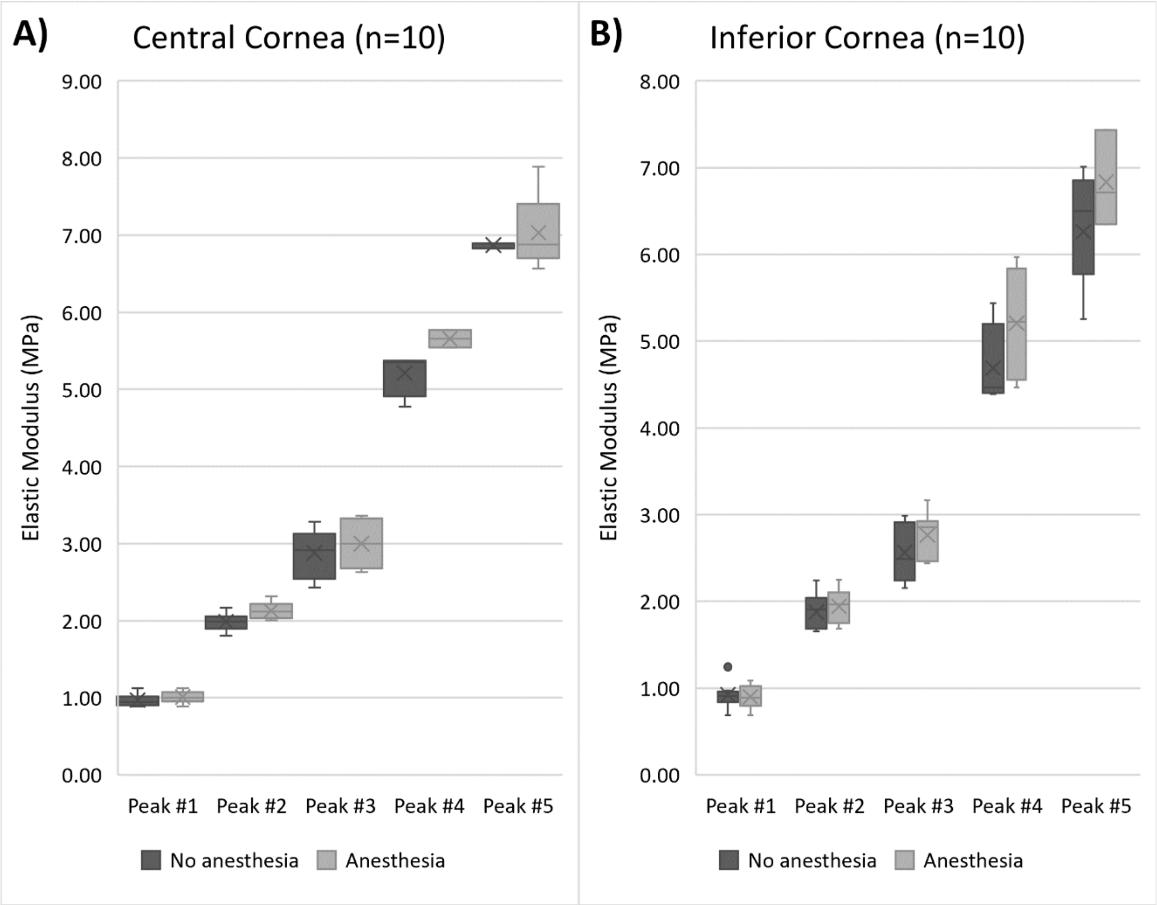

Figure S2. Elastic modulus values with and without the use of anesthesia for peaks #1-5 for (A) central and (B) inferior cornea. There was a significant difference ($p<0.05$) in the modulus value for peak #2 within the central cornea, however, the percentage difference was only 7.3%. The remaining peaks did not show a statistically significant difference ($p>0.05$ for all). The horizontal lines in the box and whisker plots represent the median values, and the boxes represent the lower and upper quartiles. The x represents the mean, and the bars represent the minimum and maximum values within 1.5 times the lower and upper quartiles. The dots represent the outlier values.
