# Supplementary material for: In Vivo Determination of the Human Corneal Elastic Modulus Using Vibrational Optical Coherence Tomography
Source: Transl Vis Sci Technol. 2022 Jul 13;11(7):11. doi: 10.1167/tvst.11.7.11 (PMC9288150; doi:10.1167/tvst.11.7.11)
Supplement: Supplement 3 [file tvst-11-7-11_s003.pdf]

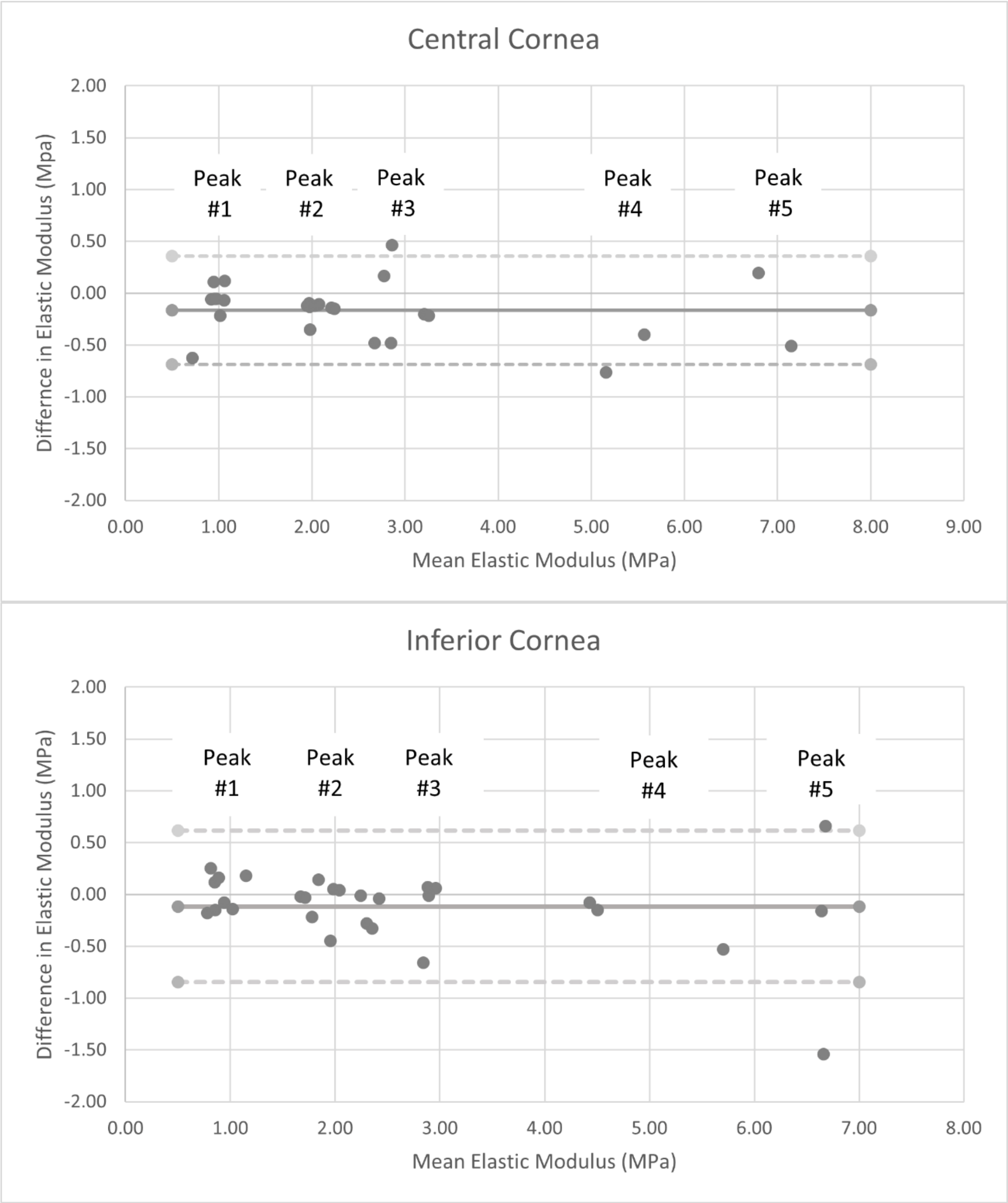

Figure S3. Differences in elastic modulus measured before and after the use of topical anesthesia. The solid line represents the mean, with upper and lower dashed lines representing the 95% confidence interval.
